# Spatial and Temporal Variation in Reproduction of a Generalist Crocodilian, *Caiman crocodilus yacare*, in a Seasonally Flooded Wetland

**DOI:** 10.1371/journal.pone.0129368

**Published:** 2015-06-24

**Authors:** Zilca Campos, Guilherme Mourão, Marcos Coutinho, William E. Magnusson, Balbina M. A. Soriano

**Affiliations:** 1 Embrapa Pantanal, Corumbá, Mato Grosso do Sul, Brazil; 2 Instituto Chico Mendes de Conservação da Biodiversidade, Lagoa Santa, Minas Gerais, Brazil; 3 Coordenação de Biodiversidade, Instituto Nacional de Pesquisas da Amazônia, Manaus, Amazonas, Brazil; BiK-F Biodiversity and Climate Research Center, GERMANY

## Abstract

We monitored the number of caiman (*Caiman crocodilus yacare*) nests in two ranches in the Brazilian Pantanal that cover an area of about 50.000 ha for 28 years (1987–2014). The number of nests was related to combinations of rainfall, water level, and number of days with temperature below 20°C, depending on the area. Most of the variation in number of nests could not be predicted by the environmental variables, but could be represented mathematically by a sine wave. We were not able to identify any external driver and suspect that the regular fluctuations may have resulted from an intrinsic population process. Presently, ranches are used as management units under the legislation for ranching Pantanal caimans. However, although some breeding females were recaptured in the area after periods of up to 21 years, most were not recaptured near nests or in general surveys of the area, suggesting that females are not strongly philopatric and that ranches do not represent isolated demographic units.

## Introduction

Reproductive traits of organisms are limited by species-specific morphological characteristics, and these lead to predictable relationships, such as between body and clutch size [1], but many species show phenotypic plasticity in reproductive strategies in response to resource availability [2–3]. Females that can respond phenotypically are expected to increase overall reproductive output in good years [4], but they may also respond behaviorally, seeking different locations to breed as conditions change. This flexibility may make it difficult to predict reproductive output from environmental predictors, because females may use different areas in different years.

Most female crocodilians appear to breed within relatively small home ranges [5–6]. Therefore, management of local demographic units, such as egg collection and maintaining hatchlings in captivity until they attain sizes less vulnerable to predators (head starting), may increase local recruitment and permit higher harvests of adults in that area for these species. This is the basis of current management of caimans in the Pantanal based on the a management-agency decree (IBAMA IN N° 61/2005). However, if adults show little philopatry, and range over wide areas, head starting hatchlings may have little effect on the local density of adult caimans. Pantanal caimans do range over wide areas [7], but data are not available on the factors that affect the intensity and distribution of nesting.

*Caiman crocodilus* is the most widespread caiman, and can be found from Mexico to Argentina. The species shows geographic variation in morphology, and individuals from different regions are often ascribed to different subspecies [8]. Individuals from the Pantanal wetlands of the Paraguay River are often allocated to a separate species, *Caiman yacare*, but there is continuous genetic integration between caimans in the Pantanal and Amazônia [9–10]. There are no consistent differences between the two taxa and they interbreed over a latitudinal range of over 10 degrees (>1000 km) [11–12]. There may be political or economic reasons to maintain the specific status of the Pantanal caiman [13]. However, as there is no way of separating the species except an arbitrary geographical limit, we use the trinomial *Caiman crocodilus yacare* as the name of the taxon we studied. Although *C*. *c*. *yacare* cannot be reliably distinguished from *Caiman crocodilus crocodilus* throughout much of its range, individuals in our study area are unequivocably attributable to *C*. *c*. *yacare*. Within its wide range, *C*. *crocodilus* can be found in different biomes and climatic conditions, and even *C*. *c*. *yacare* extends from the Amazonian tropics to temperate areas in the north of Argentina, indicating extreme genetic or behavioral adaptability. Evaluation of behavioral plasticity in caimans requires long-term studies over relatively large areas, and no such studies have been published to date.

The Brazilian Pantanal is formed by the flood plain of the Paraguay River. During the dry season, water is only found in the major rivers and isolated pools or lakes [14], but most of the 100.000,00 ha area is inundated during high water [15]. Space and food resources are extremely limited for caimans during the dry season, and many die in exceptionally dry years [16]. Water level, which is a function of the height of the Paraguay River and local rainfall, therefore determines food resources, but the Pantanal is near the southern limit of the species´ range, and low temperatures during the dry winter may also affect reproduction. Reproductive effort in *Alligator mississippiensis* is closely related to winter temperatures [17–18] and the number of days with temperature below 20°C affects onset of egg laying in *Caiman latirostris* [19], whose latitudinal range overlaps that of C. c. yacare [20]. C. c. yacare in our study site breeds at the onset of the wet season and the amount of flooding effects where caimans nest [21], but it is not known whether the number of females nesting is related to temperature or water levels.

The Pantanal has a variety of landscape elements [22–23]. Some nesting occurs around permanent water bodies, but many nests are found around temporary lakes and forest patches that are only available to most caimans during high water [21]. Nhumirim Ranch is a site of the Brazilian long-term ecological research (LTER) program [16]. During wet years, it supports over 100 lakes [24], but may dry out completely in some years [16]. Long-term studies of caiman have been undertaken on Nhumirim Ranch and the adjacent Campo Dora Ranch, which lacks the high density of lakes, but has intermittent rivers that hold water in pools even in very dry years [25–26].

The Pantanal is affected by climatic conditions in the headwaters of the rivers that feed into it, and the extent of flooding is only partly related to local rainfall [27]. The height of the Paraguay River, and hence the extent of inundation, show multiyear cycles [13, 16]. River course modifications, such as dams and manmade levees affect the extent of local flooding [28–30]. Climate change may also affect ambient temperatures in the Pantanal [31–32], and hence nesting by caimans. Evaluation of the effects of these complex, often correlated, changes requires long-term studies. The number and characteristics of caimans nesting in Nhumirim Ranch has been monitored since 1987, and less-intensive studies have been undertaken in Campo Dora Ranch in many of those years. It is not known how caimans respond to spatial and temporal differences in water availability for reproduction and this data set allowed us to evaluate factors affecting reproduction of *Caiman crocodilus yacare* in the two adjacent landscape elements.

## Material and Methods

### Study area

**Fig 1:**
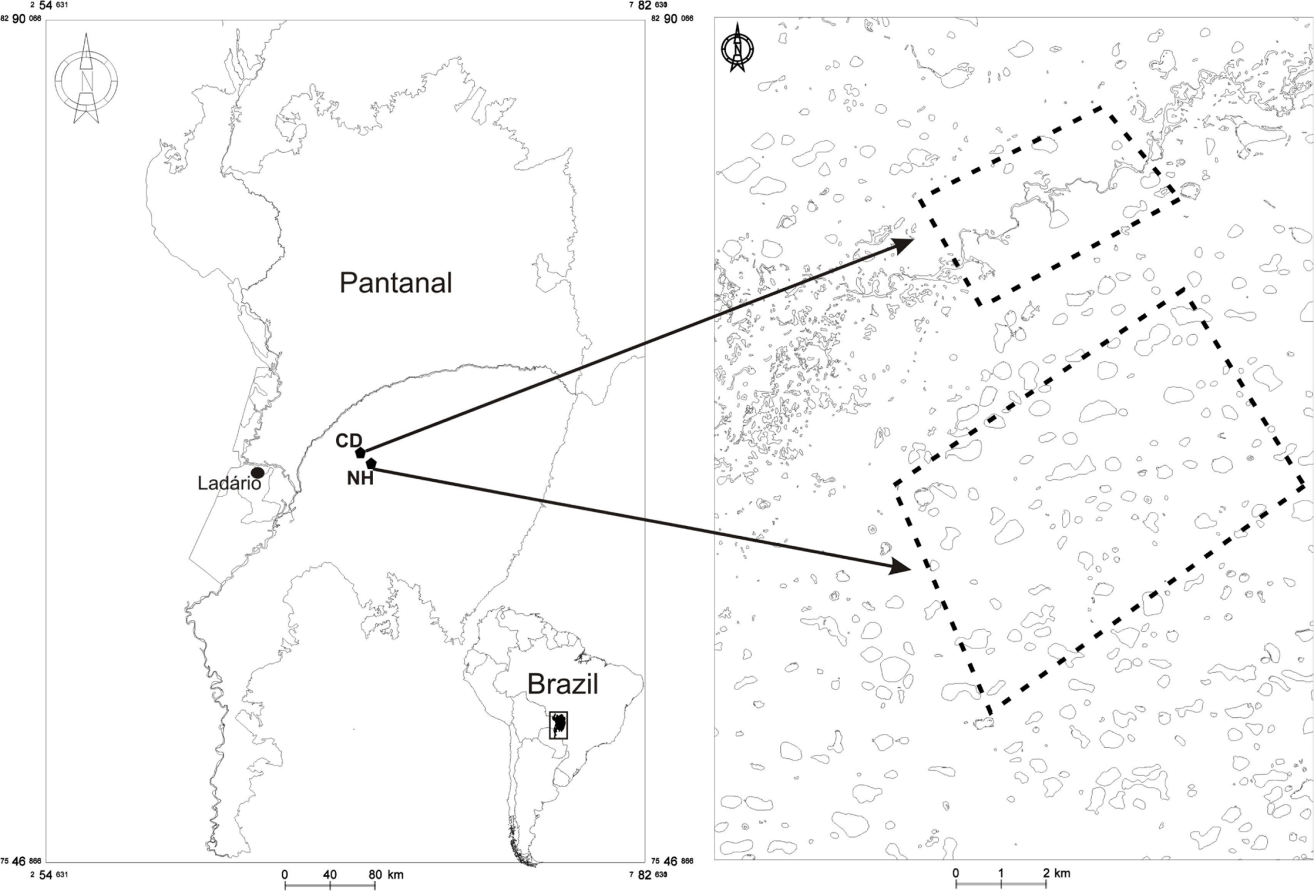
Location of the study area (CD = Campo Dora Ranch and NH = Nhumirim Ranch) in Brazilian Pantanal. Lakes in NH and intermittent river in CD. Dark symbol indicate Paraguay River, Ladário.

Nhumirim Ranch (18°59`S and 56°39´W) covers 4310 ha with around 100 lakes [24]. We also surveyed ~1000 ha around an intermittent river on Campo Dora Ranch (18°55`S and 56°39´W), which is adjacent to Nhumirim Ranch and drains to the Taquari River, Brazilian Pantanal (Fig 1). Surveys for nests were undertaken yearly on Nhumirim Ranch in January and February between 1987 and 2014. Surveys of Campo Dora were carried out in the same months in 1989–1990; 1993, 1995–2000 and 2004–2014. Both ranches have been the focus of long-term studies of caimans [25, 26, 33, 34]. Caimans regularly move been the ranches and other areas [7], so the study area does not represent a closed system for the species. Illegal hunting occurred on both ranches in the early 1980s, but hunting has been negligible since 1990 [35].

### Nest surveys

Nests were located on foot, on horseback, and using ultra-light aircraft [24]. All areas within 200m of water bodies were searched intensively each year. The species, like all members of the family Alligatoridae, only make mound nests [36]. These are easily visible if constructed in open areas, and areas of forest are limited to small patches, generally less than 50 m wide, around lakes, so we believe that we found most, if not all nests, in most of these areas each year.

Caimans nest on floating grass mats when they are available in the lakes of Nhumirim Ranch [37], and some of these cannot be surveyed effectively on foot. Nests on floating grass mats were searched for by scanning from higher areas or climbing trees beside the lakes. We surveyed these areas using an ultralight aircraft between 1989 and 2005, but the plane was not available before 1989 or after 2005. Due to the drying of the climate, floating grass mats did not form in any of the lakes we surveyed in later years (at least 10 per year), but as but as other teams surveyed the other lakes and we did not have an aircraft to check all lakes it is possible that some residual grass mats occurred in a few lakes in the period 2006–2009. Therefore, numbers of nests on Nhumirim Ranch in those years should be considered as minimum estimates. After 2010, Nhumirim became very dry and searches did not reveal any floating-grass mats capable of supporting a nest. Greater effort was expended looking for floating nests from high points in 1987 and 1988 before we obtained the ultralight, but this methodology may not be as effective as aerial search. In any case, there was no significant difference in the number of nests found on Nhumirim Ranch or any of the predictor variables we used between years in which we used an ultralight and years when none was available (Students t-test, P>0.344 in all cases). Therefore, variation in methodology would not be responsible for any of the statistically significant relationships we report.

Nesting female caimans were captured with a noose, weighed with a 50 kg spring balance (limit of reading 1kg) and snout-vent length (to the posterior edge of the cloaca) measured with a measuring tape (limit of measurement 0.1 cm). Caimans were marked by numbered plastic tags placed in the raised single tail scutes, aluminum numbered tags attached to one the interdigital membranes of the left hind leg, and/or by removing single and double tail scutes in unique combinations. They were released within 15 minutes at the point of capture. Marked caimans were also recaptured away from nests during other studies [34]. Of the 873 nests that were checked, only 5% did not contain eggs.

### Environmental variables

The number of nests found was related to the maximum height of the Paraguay River at Ladário, Mato Grosso do Sul, an index of extent of flooding in the Pantanal [16], the accumulated rainfall over the previous 12 months, which affects water availability and vegetation growth, and the number of days with minimum temperature below 20°C between May and August, which reflects temperatures unsuitable for activity during the dry season (Austral winter). Data for weather were collected in the Nhumirim Meteorological Station [38–40]. The inundated area of Nhumirim Ranch was estimated from Landsat satellite images taken in the dry season each year in the Arcview program by the in Embrapa Pantanal remote sensing laboratory. We also investigated other indices, such as the mean temperature in the dry season and water levels at other locations. However, as these resulted in qualitatively similar conclusions, we present only the analyses for the variables listed above. These alternative measures are deposited with the data we used.

### Statistical analyses

We tested for temporal autocorrelation in response and predictor variables, and in the residuals from regression analyses, using the correlog function of the pgirmess package in the R software version 2.8.0 [41]. Two-tailed probabilities for the values of the Moran statistic were derived from a permutation test for each distance-class. Autocorrelation is a correlation between values of a variable and values of the same variable at some interval of time or distance, and if present in both response and predictor variables can cause statistical artifacts [42]. We tested for serial randomness in autocorrelations using the serial randomness test [43]. The periodicity in the autocorrelation was used to select the period of a sine curve that was an empirical representation of the fluctuations in the number of females. We do not know what caused that fluctuation, but believe that it may have been an endogenous demographic processes unrelated to external variables. The period was adjusted to eliminate autocorrelation in the residuals of the multiple regression analyses. Modeling the autocorrelation process, independent of its cause, was necessary to meet the assumptions of the statistical analyses [41]. The multiple regression analyses were undertaken with the SYSTAT version 11.0 program [44].

### Research permits

The research project was approved by the Brazilian Environmental Agency (IBAMA permit N°. 017/02) and by the Chico Mendes Institute for Biodiversity Conservation (ICMBio permanent license N°.13048-1) for capture and marking caimans (relevant legislation IN N° 154/2007). All procedures followed ethical practices for animals approved by Committee on the Ethics of Animals of the Brazilian Agricultural Research Organization (Embrapa). No collection of biological material (blood, tissue etc) was used in this study. Nhumirim Ranch is owned by Embrapa. Campo Dora Ranch is private ranch, and its owners, Luís Gomes da Silva and Vicente Gomes da Silva and family, have authorized caimans research since 1987. This species was classified by International Union for Conservation of Nature (IUCN) as Lower Risk, least concern for conservation.

## Results

**Fig 2:**
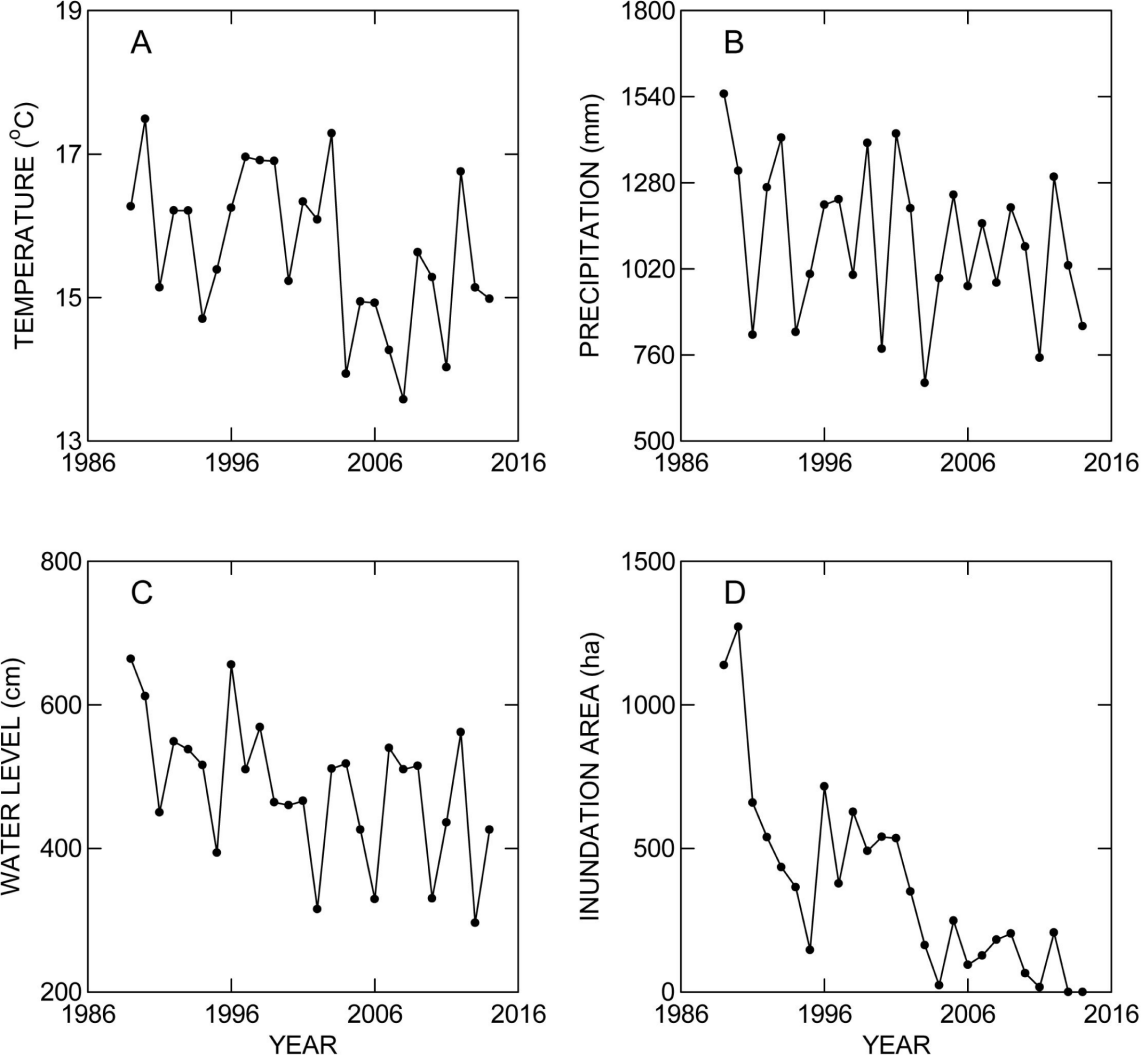
Variation in (A) minimum mean temperature (°C) between May and August, (B) Annual rainfall (mm), (C) maximum water level of the Paraguay River (m), and (D) inundated area of Nhumirim Ranch (ha) throughout the study period.

The general climate in the study area changed during the study period (S1 Table). Annual rainfall (R—mm) measured at Nhumirim Ranch (Fig 2B), maximum water level (H—m) of the Paraguay River (Fig 2C), mean minimum daily temperature (T- °C) during the dry season (Fig 2A), and maximum area inundated (A—ha) on Nhumirim Ranch (Fig 2D) decreased linearly with years since the study started (Y). All these relationships were statistically significant (R = 22607–10.74Y, N = 28, R^2^ = 0.14, P = 0.046), (H = 10190–4.85Y, N = 28, R^2^ = 0.19, P = 0.025), (T = 202.4–0.093Y, N = 29, R^2^ = 0.46, P = 0.00006), (A = 6.93–34.42Y, N = 26, R^2^ = 0.64, P = 0.000001).

**Fig 3:**
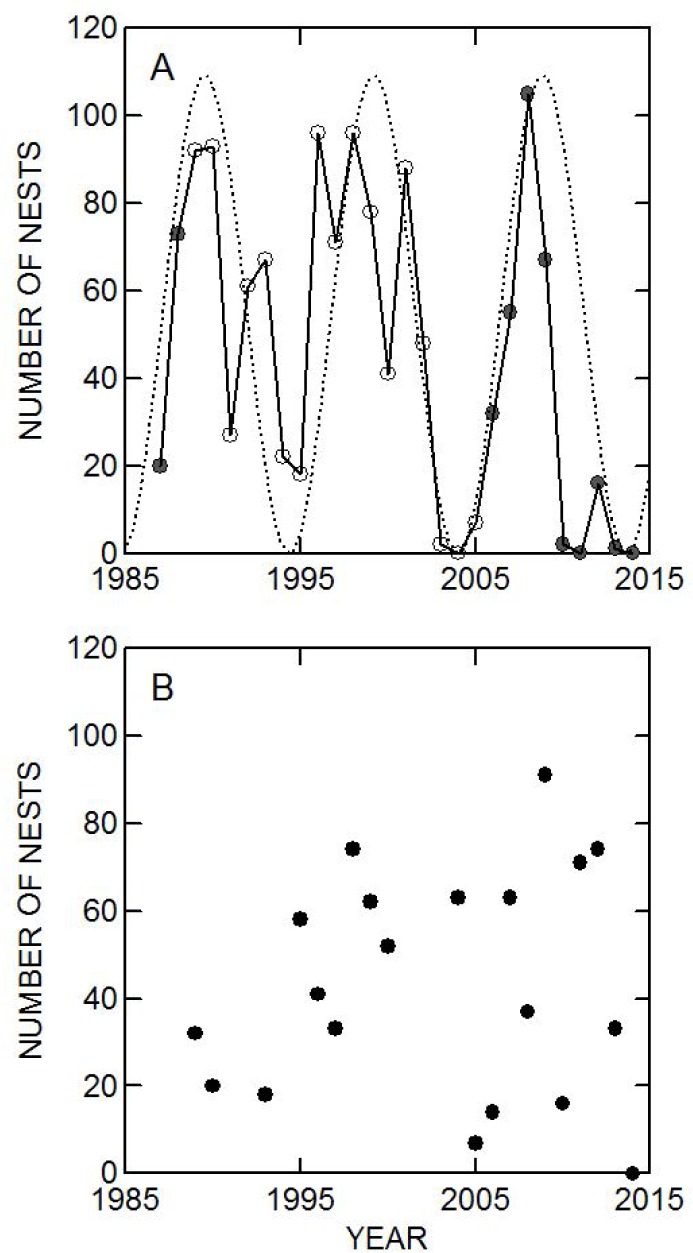
Number of nests of *Caiman crocodilus yacare* found on (A) Nhumirim Ranch and (B) Campo Dora Ranch between 1987 and 2014. Open symbols indicate years with the use of an aircraft to survey for nests on floating grass mats. Dark symbols indicate years without the use of an aircraft to survey for nests on floating grass mats. Numbers in these years may be underestimates. Solid line is the number of nests and the dotted line is the sine wave.

The number of nests on Nhumirim Ranch (Fig 3A) varied considerably among years, and there were no nests in some of the dry years after 2000. There was no consistent linear trend, but the mean number of nests on Nhumirim Ranch before 2000 (65.5, standard deviation (S) = 30.3) was significantly (Student´s t-test: t_24_ = 2.69, P = 0.013) higher than after 2000 (30.9, S = 35.1). The number of nests on Campo Dora Ranch showed no tendency to increase or decrease (Fig 3B) and there was no significant difference (Student´s t-test: t_18_ = 0.10, P = 0.92) in the mean number of nests before (42.3, S = 20.4) and after (43.4, S = 29.8) the year 2000.

**Fig 4:**
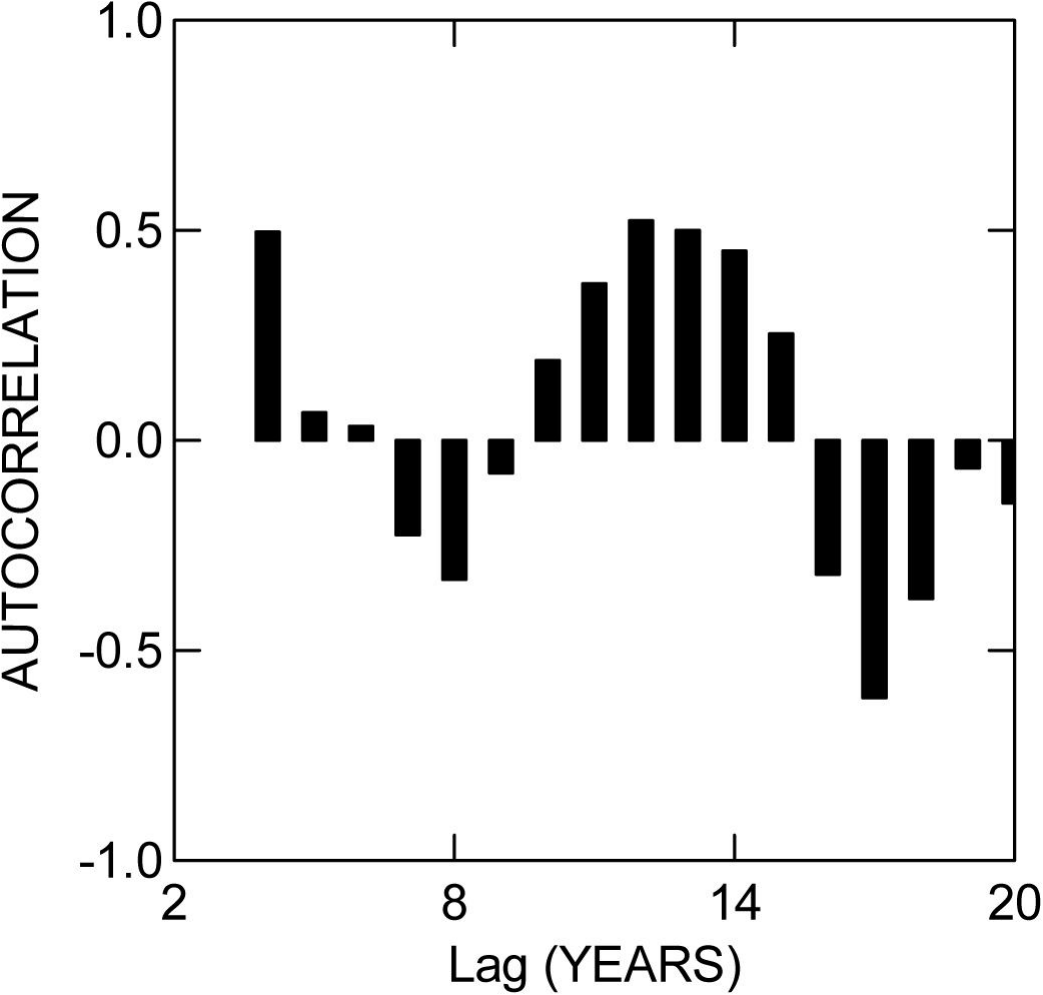
Autocorrelation in number of nests of *Caiman crocodilus yacare* on Nhumirim Ranch for lag intervals up to 20 years.

All variables measured showed autocorrelation at some lag interval (Permutation test for Moran´s I: P<0.05 in all cases). The lagged autocorrelations of number of nests on Nhumirim Ranch were not serially random (Serial Randomness test: C_20_ = 0.71, P = 0.0005), but there was no significant deviation from serial randomness for lagged autocorrelations of number of nests on Campo Dora Ranch (Serial Randomness test: C_20_<0.001, P>0.25). The pattern of autocorrelation for number of nests on Nhumirim Ranch indicated a probable cyclical pattern with a periodicity of about 9 years (Fig 4). Based on this result, the process causing the autocorrelation was modeled using a sine wave with periodicity of 9 years, and this model closely mirrored the major fluctuations in the number of nests on Nhumirim Ranch (Fig 3A).

**Fig 5:**
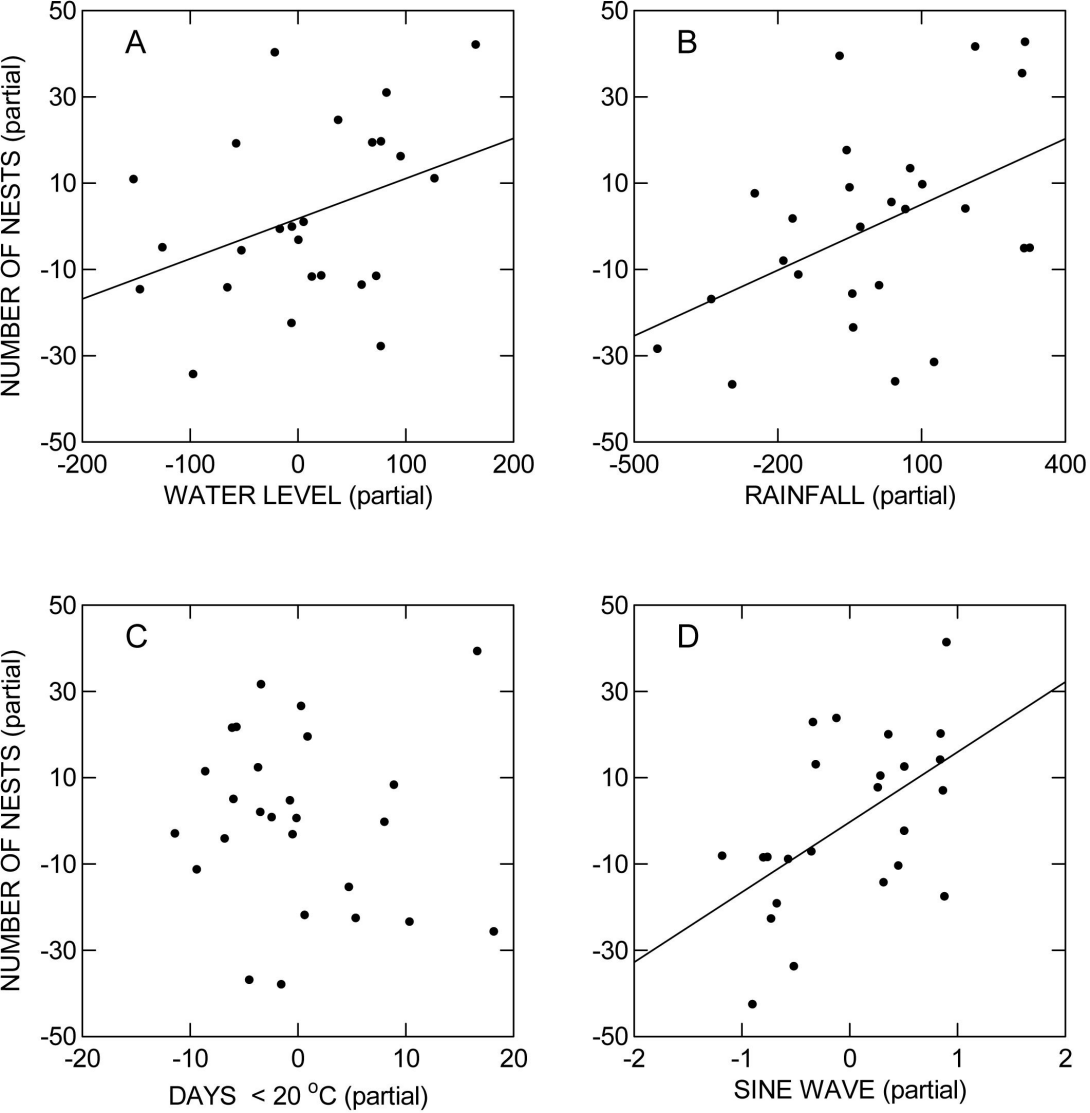
Partial regressions derived from multiple regression (see text) of (A) maximum height of the Paraguay River (m), (B) rainfall (mm) in the previous year, (C) number of days with minimum temperature below 20°C in the previous dry season, and (D) a sine wave representing endogenous demographic processes on the number of nests on Nhumirim Ranch.

Multiple regression indicated that the number of nests on Nhumirim Ranch (NN) could be predicted by a model containing height of the Paraguay River (RH—m), annual rainfall (AR—mm), number of days (ND) in the dry season with minimum temperature <20°C, and the sine wave representing the cyclical pattern (ED) (NN = -65.2 + 0.131RH + 0.051AR—0.226ND + 23.6ED, R^2^ = 0.70, F_4,21_ = 12.10, P = 0.000). RH (P = 0.017), AR (P = 0.026) and ED (P = 0.001) contributed significantly to the model, but ND (P = 0.705) did not. The number of nests increased with AR (Fig 5A), RH (Fig 5B), and ED (Fig 5E), but showed not tendency with ND (Fig 5C). Inclusion of ED in the model removed the autocorrelation in the residuals that was present when only the environmental predictors were included or ED was modeled with a period >9 years.

Multiple regression indicated that the number of nests on Campo Dora Ranch (NC) was not predicted by the same model (F_4,15_ = 1.58, P = 0.231). There was no autocorrelation in the residuals with or without inclusion of ED for data from Campo Dora.

**Fig 6:**
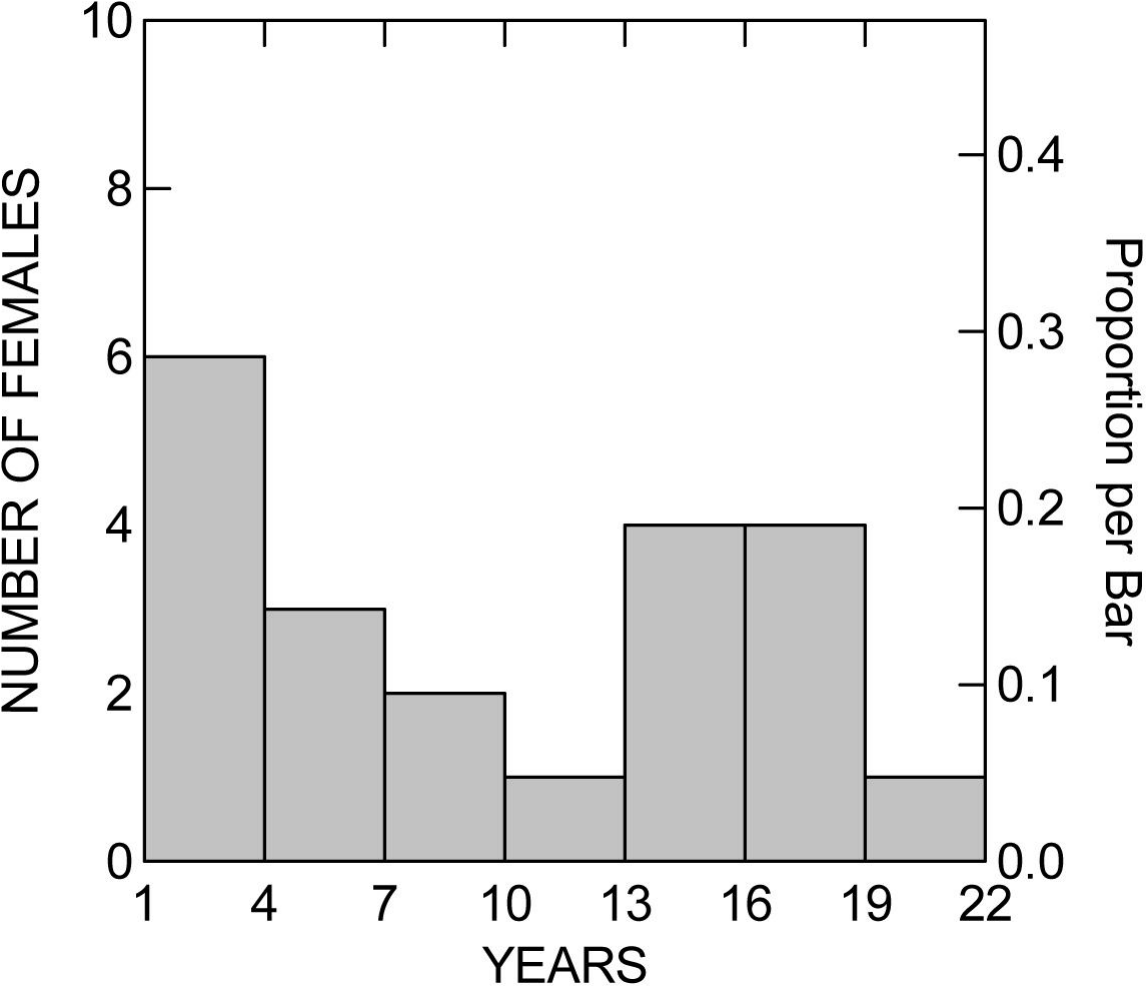
Distribution of intervals over which individual females were recaptured at nests on Nhumirim and Campo Dora ranches.

There was no statistically significant relationship between the number of nests found on Nhumirim Ranch and on Campo Dora Ranch (Pearson´s correlation: r = 0.12, P = 0.61), but the mean annual snout-vent lengths of females caught on the two ranches were positively correlated (r = 0.63, P = 0.004), indicating that that the two ranches share the same pool of nesting females in most years. Of the 364 females caught at nests, only 21 were recaptured nesting within the area, and only 7 were among the 1628 caimans captured during other studies in the area. Twenty one caimans originally captured beside nests were recaptured attending nests (S2 Table), and presumably still breeding, for periods of up to 21 years (Fig 6).

## Discussion and Conclusion

Our results are based on data collected by different observers over a 28-year period. Caiman nests are only known to be constructed within 200 m of water bodies in the area, and those areas were searched intensively each year multiple times by experienced teams. Both areas are also used intensively for cattle ranching, and the stockmen on both ranches would have alerted us if they had encountered nests in other areas. Although it is possible that a few nests were missed, and observers may have slight differences in the ability to find nests, it is unlikely that those factors affect our conclusions. There is no reason to believe that any possible observer bias was correlated with any of the environmental variables that we show are related to nesting effort. Nor is there any reason to believe that any such bias would be associated with a nine-year cycle. Any such effects would only have increased the variability and reduced the chance of encountering statistically significant relationships for the variables we analyzed.

The Pantanal is an extremely dynamic system, with many natural and human induced changes happening simultaneously, and this makes it difficult to isolate the effects of individual factors, such as the effect of hydrelectric dams on the water level of Paraguay River [28–29] and alterations in sedimentation and manmade levees on the Taquari River [30]. Our study, which covered 28 years, was undertaken during a period of continual climate change. There were linear decreases in rainfall, mean height of the Paraguay River, the area innundated on Nhumirim Ranch and the mean minimum temperatures in the dry season during the study. How much of these changes were due to decadal cycles [16], long-term climate change, and human interventions cannot be determined with our data. Although all are likely to affect nesting by caimans in the Pantanal, only nesting of caimans on Nhumirim Ranch decreased over the study period. The number on Campo Dora Ranch remained relatively constant. This may be because water levels in Campo Dora are more affected by basin-wide precipitation, which affects the major rivers, while water levels in Nhumirim are more affected by local rainfall, which declined during the study.

There are many different landscape units in the Pantanal [22–23], and we studied only parts of two. We expected them to show contrasting patterns in the number of nesting females because Nhumirim Ranch is on higher ground and is much more affected by local rainfall, whereas the intermittent rivers of Campo Dora Ranch are much more affected by overflow of the major rivers, especially the Taquari River. There was, however, no significant correlation between the number of caimans nesting on the two ranches. Therefore, fluctuations in the number of nesting caimans seem to depend more on the recruitment from remote areas and little on reciprocal movements between the adjacent ranches. However, the mean sizes of caimans varied among years, and were positively correlated between the two ranches, so the same pools of potential nesters appear to be available to both ranches each year.

Resources for caimans are likely to depend on the amount of inundated areas, and this is reflected in the positive relationship between the number of nesting caimans and the mean height of the Paraguay River for Nhumirim ranch. However, rainfall is likely to be more important for caimans on Nhumirim Ranch because the perched lakes usually do not receive water directly from the major rivers. In contrast, water levels on Campo Dora Ranch are largely independent of rainfall. Nesting increased with rainfall on Nhumirim Ranch, but did not affect nesting on Campo Dora Ranch. Perhaps caimans are more likely to move away from intermittent rivers in years with higher rainfall, but other differences between the ranches do not have such simple explanations. The number of caimans nesting was not related to the number of cold days in the dry season on either ranch.

Relationships between rainfall, temperature and nesting are expected from the natural history of the species and what is known about other crocodilians. However, most of the variation in the number of females nesting on Nhumirim Ranch followed a cyclical pattern that we could not attribute to any external factors. We did not detect any such pattern for Campo Dora Ranch, but we had much fewer data, and the longest continuous series of data collection on that ranch was only 11 years. The cycle on Nhumirim Ranch is about 9 years, which is less than some potential drivers, such as sunspots, and less than the 10–15 years estimated to be necessary for caimans in this area to attain sexual maturity [34]. We believe that it is likely to be a result of chance events causing synchrony in reproduction reinforced by demographic processes.

Random fluctuations in resource availablitiy mediated through differences in population rates of increase can cause pseudocycles that are not predictable from environmental drivers [45]. It is also possible that caimans have some behavioral mechanism to promote synchronous reproduction, analogous to masting in plants. At the moment, further speculation is unwarranted, but studies should be continued to determine whether the cycle continues, whether it occurs in other parts of the Pantanal, and whether the number of caimans nesting is related to the size structure of the pool of caimans nesting each year. The cycle could only be revealed with long-term data, illustrating the importance of long-term research sites for understanding ecological processes [46].

One adult female was captured beside a nest and recaptured beside a nest after 21 years, and many were recaptured attending nests for over a decade. Seven of the females were recaptured on a different ranch from the first capture, so it is likely that many females moved out of the study area. We do not know how many times females had nested before first capture, but the limited data indicate that females can breed over periods of several decades. This may be important to buffer numbers against the large interannual fluctuations in environmental conditions that probably strongly affect egg and hatchling survival.

Researchers often refer to the individuals of a species in their study area as a population, but this is rarely appropriate [47]. The individuals that use the two ranches we studied, which cover about 50.000 ha, are in no sense a closed population. Most frequently move large distances [7], and few females that nested in the area were recaptured during this or other studies. It appears that many are vagrants, nesting in suitable habitat wherever they happen to be during years and seasons when they are reproductively active. This is important for management options, because strategies, such as head starting hatchlings, will have little effect on the number of caimans available for harvesting on that ranch in the future. In contrast, activities designed to increase the attractiveness of a ranch for caimans, such as increasing the amount of water in the dry season and protecting forests used for nesting, which is the basis of the management program in Venezuela [48], are likely to be effective because of the large numbers of caimans continually moving across the landscape.

## Supporting Information

S1 Table Variation in annual rainfall, maximum water level, minimum temperature, number of nests in Nhumirim and Campo Dora Ranch, between 1987–2014. (PDF)

S2 Table Snout vent-length of the females reproductive and intervals between captures and recaptures. (DOCX)
