# Supplementary material for: Spatial and Temporal Variation in Reproduction of a Generalist Crocodilian, Caiman crocodilus yacare, in a Seasonally Flooded Wetland
Source: PLoS One. 2015 Jun 24;10(6):e0129368. doi: 10.1371/journal.pone.0129368 (PMC4481102; doi:10.1371/journal.pone.0129368)
Supplement: S1 Table — (PDF) [file pone.0129368.s001.pdf]

S1 Table. List of variables, annual rainfall, maximum water level, minimum temperature (ºC), number of nests in Nhumirim and Campo Dora Ranch between 1987-2014.

Year	Annual Rainfall (mm)	Water Level (cm)	Minimum Temperature (ºC)	Nhumirim Nests	Campo Dora Nests
1987	1353.30	433	17.34	73	-
1988	1282.50	499	17.51	92	-
1989	1548.00	664	16.26	93	32
1990	1316.10	612	17.48	27	20
1991	820.3	450	15.14	61	-
1992	1265.80	549	16.21	67	-
1993	1416.00	538	16.20	22	18
1994	828.7	516	14.70	18	-
1995	10004.30	394	15.39	96	58
1996	1213.20	656	16.24	71	41
1997	1229.60	510	16.96	96	33
1998	1001.70	569	16.91	78	74
1999	1400.20	464	16.90	41	62
2000	778.1	460	15.23	88	52
2001	1428.20	466	16.33	48	-
2002	1202.50	315	16.08	2	-
2003	675.7	511	17.28	0	-
2004	991.8	518	13.93	7	63
2005	1243.20	426	14.94	32	7
2006	967.7	329	14.92	55	14
2007	1156.40	540	14.26	105	63
2008	978.2	510	13.58	67	37
2009	1204.60	515	15.63	2	91
2010	1087.00	330	15.28	0	16
2011	751.5	436	14.02	16	71
2012	297.90	562	16.75	1	74
2013	1030.30	296	15.13	0	33
2014	846.4	426	14.98	0	0
