# Supplementary material for: Spatial and Temporal Variation in Reproduction of a Generalist Crocodilian, Caiman crocodilus yacare, in a Seasonally Flooded Wetland
Source: PLoS One. 2015 Jun 24;10(6):e0129368. doi: 10.1371/journal.pone.0129368 (PMC4481102; doi:10.1371/journal.pone.0129368)
Supplement: S2 Table — (DOCX) [file pone.0129368.s002.docx]

**S2. Snout vent- length (SVL, cm) of the females**

**reproductive (ID) and intervals between captures and**

**recaptures.**

| ID | SVL_1 | SVL_2 | Days | Years |
| --- | --- | --- | --- | --- |
| 204 | 63.6 | 82.1 | 678 | 1.85 |
| 518 | 71 | 83.5 | 1887 | 5.16 |
| 519 | 48.5 | 89.5 | 2375 | 6.50 |
| 520 | 72.0 | 84.0 | 5912 | 16.19 |
| 521 | 52.7 | 85.0 | 5246 | 14.37 |
| 523 | 31.4 | 88.5 | 5919 | 16.21 |
| 527 | 89.5 | 89.5 | 2967 | 8.12 |
| 529 | 75.0 | 81.0 | 3003 | 8.22 |
| 532 | 12.4 | 84.5 | 6509 | 17.83 |
| 533 | 61.8 | 91.5 | 6334 | 17.35 |
| 557 | 83.5 | 87.0 | 4017 | 11.00 |
| 559 | 81.0 | 81.0 | 1046 | 2.86 |
| 560 | 84.0 | 84.0 | 1822 | 4.99 |
| 561 | 80.5 | 80.5 | 734 | 2.01 |
| 564 | 76.5 | 76.5 | 1092 | 2.99 |
| 566 | 90.5 | 90.5 | 733 | 2.00 |
| 578 | 80.5 | 83.0 | 5109 | 13.99 |
| 587 | 82.0 | 90.0 | 5221 | 14.30 |
| 614 | 84.5 | 84.5 | 956 | 2.61 |
| 635 | 35.0 | 85.0 | 7736 | 21.19 |
| 636 | 82.5 | 83.0 | 5109 | 13.99 |
